# (Acetyl­acetonato-κ^2^ *O*,*O*′)carbon­yl[dicyclo­hex­yl(2,6-diisopropyl­phen­yl)phosphane-κ*P*]rhodium(I)

**DOI:** 10.1107/S1600536812018831

**Published:** 2012-05-05

**Authors:** Wade L. Davis, Sfiso D. Mathobela, Reinout Meijboom

**Affiliations:** a Research Center for Synthesis and Catalysis, Department of Chemistry, University of Johannesburg (APK Campus), PO Box 524, Auckland Park, Johannesburg 2006, South Africa

## Abstract

In the title compound, [Rh(C_5_H_7_O_2_){C_12_H_17_P(C_6_H_11_)_2_}(CO)], the Rh^I^ atom is coordinated by one carbonyl C, one P and two O atoms, forming a slighlty distorted square-planar configuration.

## Related literature

For background literature on the catalytic activity of rhodium–phosphine compounds, see Moloy & Wegman (1989 ▶); Nozaki *et al.* (1997 ▶); Ocando-Mavarez *et al.* (2003 ▶); Hayashi & Yamasaki (2003 ▶); Erasmus & Conradie (2011 ▶). For related rhodium compounds, see: Riihimaki *et al.* (2003 ▶); Brink *et al.* (2007 ▶); Davis & Meijboom (2011 ▶).
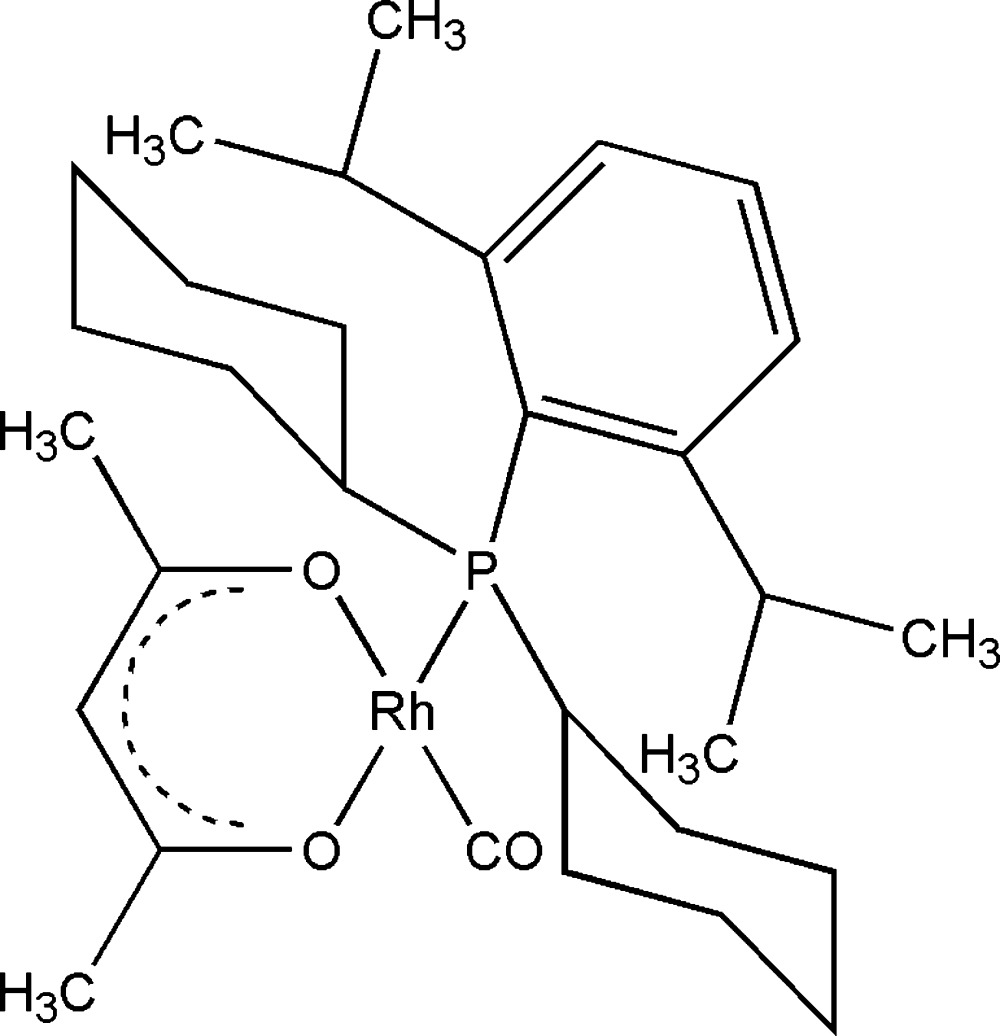


## Experimental

### 

#### Crystal data

[Rh(C_5_H_7_O_2_)(C_24_H_39_P)(CO)]
*M* *_r_* = 588.55
Monoclinic, 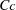

*a* = 16.750 (2) Å
*b* = 9.7334 (13) Å
*c* = 19.385 (3) Å
β = 111.669 (3)°
*V* = 2937.1 (7) Å^3^
*Z* = 4
Mo *K*α radiation
μ = 0.66 mm^−1^
*T* = 100 K
0.29 × 0.23 × 0.22 mm

#### Data collection

Bruker APEX DUO 4K-CCD diffractometer
Absorption correction: multi-scan (*SADABS*; Bruker, 2008 ▶) *T*_min_ = 0.553, *T*_max_ = 0.746
14224 measured reflections
6007 independent reflections
5516 reflections with *I* > 2σ(*I*)
*R*_int_ = 0.049

#### Refinement

*R*[*F* ^2^ > 2σ(*F* ^2^)] = 0.043
*wR*(*F* ^2^) = 0.105
*S* = 1.05
6007 reflections
322 parameters
2 restraints
H-atom parameters constrained
Δρ_max_ = 1.85 e Å^−3^
Δρ_min_ = −1.42 e Å^−3^
Absolute structure: Flack (1983 ▶), 2437 Friedel pairs
Flack parameter: −0.03 (3)

### 

Data collection: *APEX2* (Bruker, 2010 ▶); cell refinement: *SAINT* (Bruker, 2008 ▶); data reduction: *SAINT* and *XPREP* (Bruker, 2008 ▶); program(s) used to solve structure: *SIR97* (Altomare *et al.*, 1999 ▶); program(s) used to refine structure: *SHELXL97* (Sheldrick, 2008 ▶); molecular graphics: *DIAMOND* (Brandenburg & Putz, 2005 ▶); software used to prepare material for publication: *publCIF* (Westrip, 2010 ▶) and *WinGX* (Farrugia, 1999 ▶).

## Supplementary Material

Crystal structure: contains datablock(s) global, I. DOI: 10.1107/S1600536812018831/aa2053sup1.cif

Structure factors: contains datablock(s) I. DOI: 10.1107/S1600536812018831/aa2053Isup2.hkl

Additional supplementary materials:  crystallographic information; 3D view; checkCIF report

## Figures and Tables

**Fig. 1.**
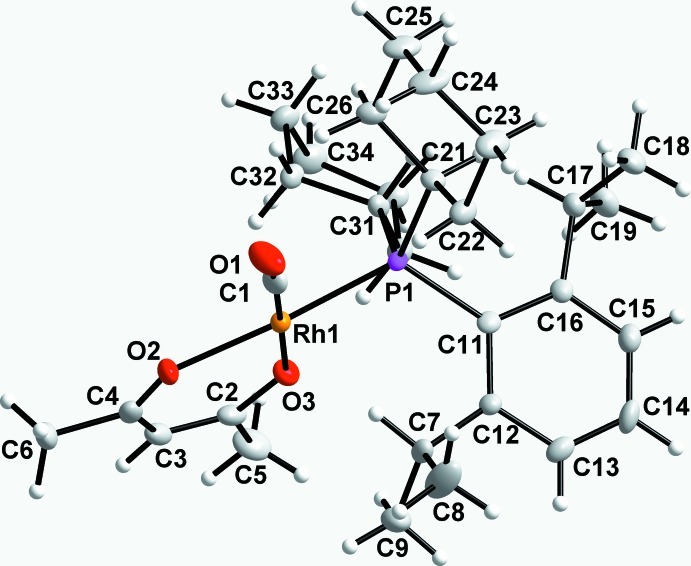
Molecular structure of the title compound, showing the atom numbering system. Displacement ellipsoids are drawn at the 50% probability level. For the C atoms in rings; the first digit indicates ring number and the second digit indicates the position of the atom in the ring.

## supplementary crystallographic information

### Comment

Transition metal complexes bearing functionalized phosphines are of interest due to their potential catalytic properties (Ocando-Mavarez *et al.*, 2003). These complexes are used with various chiral ligands in the process of highly enantioselective hydroformylation reactions (Nozaki *et al.*, 1997). Studies illustrating the catalytic importance of rhodium(I) square-planar moieties have been conducted on rhodium mono- and di-phosphane complexes containing the symmetrical bidentate ligand, acac (acac = acetylacetonate) (Moloy & Wegman, 1989; Erasmus & Conradie, 2011) as well as rhodium-catalyzed asymmetric 1,4-addition (Hayashi & Yamasaki, 2003). This work is part of an ongoing investigation aimed at determing the steric effects induced by various phosphine ligands on a rhodium(I) metal centre.

The title compound, [Rh(acac)(CO){C_12_H_17_P(C_6_H_11_)_2_}] (acac = acetylacetonate), crystallizes in the non-centrosymmetric monoclinic space group, *C c* (*Z*=4). The Rh(I) atom has a slightly distorted square-planar geometric coordination (see Fig. 1), illustrated by C1—Rh1—P1 and O2—Rh1—O3 angles of 94.54 (1)° and 89.37 (1)°, respectively, deviating from the ideal 90° right angle. A slightly asymmetric coordination of the acac ligand is observed, whereby the Rh1—O2 distance (2.083 (3) Å) is longer than that for Rh1—O3 (2.059 (3) Å), which may be attributed to a *trans* influence of the phosphane ligand. The steric demand of the phosphane ligand is indicated by the smaller O3—Rh1—P1 angle, (86.64 (9)°), compared to that of the carbonyl ligand, O2—Rh1—C1 (94.97 (1)°). All geometric parameters are similar to previous reported complexes of the general formula [Rh(acac)(CO)*L*]; *L* = tertiary phosphane ligand (Davis & Meijboom, 2011; Brink *et al.*, 2007; Riihimaki *et al.*, 2003).

### Experimental

A solution of [Rh(acac)(CO)_2_] (42.2 mg, 0.16 mmol) in acetone (5 ml) was slowly added to a solution of C_12_H_17_P(C_6_H_11_)_2_ (64.5 mg, 0.18 mmol) in acetone (5 ml). Slow evaporation of the solvent afforded the title compound as yellow crystals. Spectroscopic analysis: ^31^P NMR (CDCl_3_, 162 MHz, p.p.m.): 47.5 [d, ^1^J(Rh—P)= 165.7 Hz]; IR (CH_2_Cl_2_) ν(CO): 1959.2 cm^-1^.

### Refinement

All H atoms were placed in geometrically idealized positions (C—H = 0.95–1.00) and constrained to ride on their parent atoms, with *U*_iso_(H) = 1.2*U*_eq_(C) for aromatic, methine and methylene H atoms, and *U*_iso_(H) = 1.5*U*_eq_(C) for methyl H atoms respectively. Methyl torsion angles were refined from electron density. Friedel Pairs = 2437.

### Crystal data

**Table:**

[Rh(C_5_H_7_O_2_)(C_24_H_39_P)(CO)]	*F*(000) = 1240
*M**_r_* = 588.55	*D*_x_ = 1.331 Mg m^−^^3^
Monoclinic, *C**c*	Mo *K*α radiation, λ = 0.71073 Å
Hall symbol: C -2yc	Cell parameters from 5131 reflections
*a* = 16.750 (2) Å	θ = 2.5–27.6°
*b* = 9.7334 (13) Å	µ = 0.66 mm^−^^1^
*c* = 19.385 (3) Å	*T* = 100 K
β = 111.669 (3)°	Cubic, yellow
*V* = 2937.1 (7) Å^3^	0.29 × 0.23 × 0.22 mm
*Z* = 4	

### Data collection

**Table:**

Bruker APEX DUO 4K-CCD diffractometer	6007 independent reflections
Radiation source: sealed tube	5516 reflections with *I* > 2σ(*I*)
Graphite monochromator	*R*_int_ = 0.049
Detector resolution: 8.4 pixels mm^-1^	θ_max_ = 28.2°, θ_min_ = 2.5°
φ and ω scans	*h* = −21→22
Absorption correction: multi-scan (*SADABS*; Bruker, 2008)	*k* = −12→12
*T*_min_ = 0.553, *T*_max_ = 0.746	*l* = −25→24
14224 measured reflections	

### Refinement

**Table:**

Refinement on *F*^2^	Secondary atom site location: difference Fourier map
Least-squares matrix: full	Hydrogen site location: inferred from neighbouring sites
*R*[*F*^2^ > 2σ(*F*^2^)] = 0.043	H-atom parameters constrained
*wR*(*F*^2^) = 0.105	*w* = 1/[σ^2^(*F*_o_^2^) + (0.0588*P*)^2^] where *P* = (*F*_o_^2^ + 2*F*_c_^2^)/3
*S* = 1.05	(Δ/σ)_max_ < 0.001
6007 reflections	Δρ_max_ = 1.85 e Å^−^^3^
322 parameters	Δρ_min_ = −1.42 e Å^−^^3^
2 restraints	Absolute structure: Flack (1983), 2437 Friedel pairs
Primary atom site location: structure-invariant direct methods	Flack parameter: −0.03 (3)

### Special details

**Table:**

Experimental. The intensity data was collected on a Bruker Apex DUO 4 K CCD diffractometer using an exposure time of 2 s/frame. A total of 1125 frames were collected with a frame width of 0.5° covering up to θ = 28.18° with 99.1% completeness accomplished.
Geometry. All e.s.d.'s (except the e.s.d. in the dihedral angle between two l.s. planes) are estimated using the full covariance matrix. The cell e.s.d.'s are taken into account individually in the estimation of e.s.d.'s in distances, angles and torsion angles; correlations between e.s.d.'s in cell parameters are only used when they are defined by crystal symmetry. An approximate (isotropic) treatment of cell e.s.d.'s is used for estimating e.s.d.'s involving l.s. planes.
Refinement. Refinement of *F*^2^ against ALL reflections. The weighted *R*-factor *wR* and goodness of fit *S* are based on *F*^2^, conventional *R*-factors *R* are based on *F*, with *F* set to zero for negative *F*^2^. The threshold expression of *F*^2^ > σ(*F*^2^) is used only for calculating *R*-factors(gt) *etc*. and is not relevant to the choice of reflections for refinement. *R*-factors based on *F*^2^ are statistically about twice as large as those based on *F*, and *R*- factors based on ALL data will be even larger.

### Fractional atomic coordinates and isotropic or equivalent isotropic displacement parameters (Å^2^)

**Table:**

	*x*	*y*	*z*	*U*_iso_*/*U*_eq_	
Rh1	1.04568 (3)	0.50680 (3)	0.55184 (3)	0.01467 (8)	
P1	0.96331 (6)	0.64795 (10)	0.45914 (6)	0.0128 (2)	
O1	0.9055 (2)	0.4101 (4)	0.5979 (2)	0.0355 (9)	
O3	1.14853 (18)	0.5725 (3)	0.52656 (17)	0.0212 (6)	
O2	1.12596 (19)	0.3671 (3)	0.62714 (18)	0.0223 (7)	
C1	0.9568 (3)	0.4502 (5)	0.5774 (2)	0.0230 (9)	
C31	0.9846 (2)	0.5943 (4)	0.3744 (2)	0.0151 (8)	
H2	0.9303	0.6115	0.3309	0.018*	
C36	1.0561 (3)	0.6742 (4)	0.3596 (2)	0.0187 (8)	
H3A	1.0459	0.7742	0.3609	0.022*	
H3B	1.1125	0.6528	0.3987	0.022*	
C35	1.0568 (3)	0.6340 (5)	0.2839 (2)	0.0212 (9)	
H4A	1.1039	0.6833	0.2753	0.025*	
H4B	1.0019	0.6625	0.2449	0.025*	
C34	1.0688 (3)	0.4798 (5)	0.2781 (3)	0.0271 (10)	
H5A	1.1272	0.4535	0.312	0.033*	
H5B	1.0636	0.457	0.2268	0.033*	
C33	1.0022 (3)	0.3972 (5)	0.2977 (3)	0.0225 (9)	
H6A	0.9442	0.4137	0.2598	0.027*	
H6B	1.0149	0.2979	0.2977	0.027*	
C32	1.0034 (3)	0.4391 (4)	0.3742 (2)	0.0177 (8)	
H7A	1.0603	0.4184	0.4126	0.021*	
H7B	0.9595	0.3862	0.3859	0.021*	
C11	0.9794 (2)	0.8374 (4)	0.4696 (2)	0.0151 (8)	
C12	1.0292 (2)	0.8964 (4)	0.5403 (2)	0.0157 (8)	
C7	1.0743 (3)	0.8194 (4)	0.6126 (2)	0.0208 (9)	
H10	1.0627	0.7193	0.6019	0.025*	
C9	1.1720 (3)	0.8394 (5)	0.6400 (3)	0.0383 (13)	
H11A	1.1927	0.8107	0.6011	0.057*	
H11B	1.1998	0.7837	0.6845	0.057*	
H11C	1.1859	0.9365	0.6518	0.057*	
C8	1.0383 (4)	0.8585 (5)	0.6715 (3)	0.0405 (13)	
H12A	1.0432	0.958	0.6796	0.061*	
H12B	1.0708	0.811	0.718	0.061*	
H12C	0.9778	0.8315	0.6548	0.061*	
C13	1.0382 (4)	1.0397 (4)	0.5487 (4)	0.0209 (9)	
H13	1.0712	1.0772	0.5959	0.025*	
C14	0.9997 (3)	1.1268 (5)	0.4895 (3)	0.0245 (10)	
H14	1.0064	1.2234	0.4961	0.029*	
C15	0.9518 (3)	1.0737 (5)	0.4209 (3)	0.0218 (9)	
H15	0.9255	1.1344	0.3805	0.026*	
C16	0.9410 (2)	0.9319 (4)	0.4096 (2)	0.0161 (8)	
C17	0.8849 (3)	0.8926 (4)	0.3296 (2)	0.0203 (9)	
H17	0.885	0.7902	0.3256	0.024*	
C19	0.9197 (3)	0.9524 (5)	0.2727 (3)	0.0269 (10)	
H18A	0.9139	1.0527	0.2715	0.04*	
H18B	0.887	0.9148	0.2234	0.04*	
H18C	0.9805	0.9278	0.287	0.04*	
C18	0.7919 (3)	0.9396 (5)	0.3118 (3)	0.0273 (10)	
H19A	0.7689	0.8945	0.3457	0.041*	
H19B	0.7568	0.9148	0.2605	0.041*	
H19C	0.7906	1.0394	0.3178	0.041*	
C21	0.8441 (2)	0.6307 (4)	0.4276 (2)	0.0165 (8)	
H20	0.8194	0.6898	0.3824	0.02*	
C22	0.8091 (3)	0.6873 (4)	0.4847 (3)	0.0220 (9)	
H21A	0.8251	0.7853	0.4943	0.026*	
H21B	0.8353	0.6365	0.532	0.026*	
C23	0.7110 (3)	0.6732 (5)	0.4567 (3)	0.0290 (11)	
H22A	0.6905	0.7054	0.4956	0.035*	
H22B	0.6848	0.7323	0.4125	0.035*	
C24	0.6826 (3)	0.5260 (5)	0.4366 (3)	0.0307 (11)	
H23A	0.6191	0.521	0.4179	0.037*	
H23B	0.7056	0.4676	0.4814	0.037*	
C25	0.7146 (3)	0.4728 (5)	0.3773 (3)	0.0282 (11)	
H24A	0.6876	0.5266	0.3311	0.034*	
H24B	0.6972	0.3756	0.3661	0.034*	
C26	0.8128 (3)	0.4839 (4)	0.4030 (3)	0.0216 (9)	
H25A	0.8397	0.42	0.4449	0.026*	
H25B	0.831	0.4564	0.3619	0.026*	
C2	1.2259 (3)	0.5256 (5)	0.5547 (3)	0.0224 (9)	
C5	1.2883 (3)	0.5932 (6)	0.5257 (3)	0.0346 (12)	
H27A	1.2702	0.576	0.4723	0.052*	
H27B	1.3458	0.5551	0.5513	0.052*	
H27C	1.2893	0.6924	0.5346	0.052*	
C3	1.2540 (3)	0.4204 (5)	0.6063 (2)	0.0245 (10)	
H28	1.3128	0.3949	0.6212	0.029*	
C4	1.2047 (3)	0.3483 (4)	0.6385 (2)	0.0238 (9)	
C6	1.2468 (3)	0.2315 (5)	0.6923 (3)	0.0353 (12)	
H30A	1.2111	0.2084	0.7209	0.053*	
H30B	1.3038	0.2604	0.7262	0.053*	
H30C	1.2523	0.1507	0.6642	0.053*	

### Atomic displacement parameters (Å^2^)

**Table:**

	*U*^11^	*U*^22^	*U*^33^	*U*^12^	*U*^13^	*U*^23^
Rh1	0.01337 (12)	0.01245 (13)	0.01730 (13)	−0.00060 (14)	0.00463 (9)	0.00048 (14)
P1	0.0102 (4)	0.0110 (4)	0.0173 (5)	−0.0018 (4)	0.0051 (4)	−0.0003 (4)
O1	0.0222 (16)	0.046 (2)	0.0360 (19)	−0.0074 (16)	0.0083 (15)	0.0175 (16)
O3	0.0128 (14)	0.0242 (16)	0.0269 (16)	0.0011 (12)	0.0076 (12)	0.0011 (13)
O2	0.0182 (15)	0.0164 (14)	0.0269 (17)	0.0012 (12)	0.0020 (13)	0.0057 (12)
C1	0.026 (2)	0.021 (2)	0.019 (2)	−0.0007 (19)	0.0043 (18)	0.0068 (17)
C31	0.0133 (17)	0.0171 (18)	0.0155 (19)	0.0034 (15)	0.0060 (15)	−0.0016 (14)
C36	0.0183 (19)	0.0192 (19)	0.020 (2)	−0.0018 (16)	0.0091 (16)	0.0009 (16)
C35	0.016 (2)	0.029 (2)	0.023 (2)	−0.0042 (18)	0.0127 (17)	−0.0004 (18)
C34	0.022 (2)	0.036 (3)	0.029 (2)	0.0006 (19)	0.016 (2)	−0.0075 (19)
C33	0.021 (2)	0.022 (2)	0.027 (2)	−0.0014 (18)	0.0114 (18)	−0.0071 (17)
C32	0.0127 (17)	0.0171 (19)	0.026 (2)	0.0004 (15)	0.0096 (16)	−0.0027 (17)
C11	0.0090 (18)	0.017 (2)	0.021 (2)	−0.0017 (15)	0.0078 (16)	−0.0016 (16)
C12	0.013 (2)	0.0143 (17)	0.019 (2)	−0.0025 (14)	0.0056 (17)	−0.0003 (15)
C7	0.028 (2)	0.014 (2)	0.016 (2)	0.0003 (18)	0.0030 (17)	0.0014 (16)
C9	0.028 (2)	0.028 (3)	0.042 (3)	−0.005 (2)	−0.008 (2)	0.000 (2)
C8	0.075 (4)	0.029 (3)	0.024 (2)	−0.007 (3)	0.025 (3)	−0.002 (2)
C13	0.020 (2)	0.0188 (17)	0.028 (2)	−0.004 (2)	0.0135 (19)	−0.007 (3)
C14	0.030 (2)	0.0125 (19)	0.036 (3)	0.0042 (18)	0.018 (2)	0.0016 (17)
C15	0.019 (2)	0.017 (2)	0.030 (2)	0.0021 (17)	0.0097 (18)	0.0032 (17)
C16	0.0118 (17)	0.0167 (19)	0.021 (2)	−0.0008 (15)	0.0073 (16)	0.0013 (16)
C17	0.0180 (19)	0.018 (2)	0.022 (2)	0.0019 (16)	0.0035 (16)	0.0009 (16)
C19	0.025 (2)	0.033 (2)	0.022 (2)	0.0050 (19)	0.0070 (19)	0.0047 (19)
C18	0.017 (2)	0.029 (2)	0.030 (2)	0.0018 (18)	0.0011 (18)	0.001 (2)
C21	0.0100 (17)	0.020 (2)	0.0190 (19)	−0.0015 (15)	0.0050 (15)	0.0000 (15)
C22	0.0152 (18)	0.021 (2)	0.031 (2)	−0.0007 (16)	0.0100 (17)	−0.0059 (17)
C23	0.0135 (19)	0.033 (3)	0.043 (3)	−0.0033 (18)	0.0138 (19)	−0.010 (2)
C24	0.019 (2)	0.040 (3)	0.039 (3)	−0.0161 (19)	0.019 (2)	−0.017 (2)
C25	0.015 (2)	0.038 (3)	0.033 (3)	−0.0107 (18)	0.011 (2)	−0.013 (2)
C26	0.017 (2)	0.027 (2)	0.021 (2)	−0.0037 (17)	0.0073 (17)	−0.0056 (16)
C2	0.0127 (19)	0.026 (2)	0.026 (2)	−0.0012 (16)	0.0035 (17)	−0.0091 (17)
C5	0.015 (2)	0.051 (3)	0.039 (3)	0.001 (2)	0.011 (2)	−0.002 (2)
C3	0.0144 (19)	0.026 (2)	0.028 (2)	0.0031 (17)	0.0012 (17)	−0.0081 (18)
C4	0.027 (2)	0.018 (2)	0.019 (2)	0.0032 (17)	0.0005 (17)	−0.0056 (16)
C6	0.027 (2)	0.020 (2)	0.042 (3)	0.0058 (19)	−0.007 (2)	0.001 (2)

### Geometric parameters (Å, º)

**Table:**

Rh1—C1	1.820 (5)	C14—C15	1.374 (7)
Rh1—O3	2.059 (3)	C14—H14	0.95
Rh1—O2	2.083 (3)	C15—C16	1.399 (6)
Rh1—P1	2.2780 (12)	C15—H15	0.95
P1—C11	1.864 (4)	C16—C17	1.536 (6)
P1—C21	1.868 (4)	C17—C18	1.536 (6)
P1—C31	1.879 (4)	C17—C19	1.539 (7)
O1—C1	1.141 (6)	C17—H17	1
O3—C2	1.289 (5)	C19—H18A	0.98
O2—C4	1.268 (5)	C19—H18B	0.98
C31—C36	1.542 (6)	C19—H18C	0.98
C31—C32	1.543 (6)	C18—H19A	0.98
C31—H2	1	C18—H19B	0.98
C36—C35	1.523 (6)	C18—H19C	0.98
C36—H3A	0.99	C21—C22	1.534 (6)
C36—H3B	0.99	C21—C26	1.536 (6)
C35—C34	1.525 (6)	C21—H20	1
C35—H4A	0.99	C22—C23	1.534 (5)
C35—H4B	0.99	C22—H21A	0.99
C34—C33	1.533 (7)	C22—H21B	0.99
C34—H5A	0.99	C23—C24	1.514 (6)
C34—H5B	0.99	C23—H22A	0.99
C33—C32	1.531 (6)	C23—H22B	0.99
C33—H6A	0.99	C24—C25	1.527 (7)
C33—H6B	0.99	C24—H23A	0.99
C32—H7A	0.99	C24—H23B	0.99
C32—H7B	0.99	C25—C26	1.537 (6)
C11—C12	1.435 (5)	C25—H24A	0.99
C11—C16	1.435 (6)	C25—H24B	0.99
C12—C13	1.405 (5)	C26—H25A	0.99
C12—C7	1.522 (6)	C26—H25B	0.99
C7—C8	1.523 (7)	C2—C3	1.387 (7)
C7—C9	1.535 (7)	C2—C5	1.508 (7)
C7—H10	1	C5—H27A	0.98
C9—H11A	0.98	C5—H27B	0.98
C9—H11B	0.98	C5—H27C	0.98
C9—H11C	0.98	C3—C4	1.396 (7)
C8—H12A	0.98	C3—H28	0.95
C8—H12B	0.98	C4—C6	1.527 (6)
C8—H12C	0.98	C6—H30A	0.98
C13—C14	1.380 (8)	C6—H30B	0.98
C13—H13	0.95	C6—H30C	0.98
			
C1—Rh1—O3	178.09 (18)	C14—C15—C16	121.2 (4)
C1—Rh1—O2	89.60 (17)	C14—C15—H15	119.4
O3—Rh1—O2	89.37 (12)	C16—C15—H15	119.4
C1—Rh1—P1	94.54 (14)	C15—C16—C11	120.8 (4)
O3—Rh1—P1	86.64 (9)	C15—C16—C17	113.5 (3)
O2—Rh1—P1	173.12 (11)	C11—C16—C17	125.6 (4)
C11—P1—C21	102.61 (18)	C16—C17—C18	110.1 (4)
C11—P1—C31	107.71 (19)	C16—C17—C19	112.2 (4)
C21—P1—C31	102.32 (18)	C18—C17—C19	110.4 (4)
C11—P1—Rh1	119.33 (13)	C16—C17—H17	108
C21—P1—Rh1	117.86 (14)	C18—C17—H17	108
C31—P1—Rh1	105.47 (13)	C19—C17—H17	108
C2—O3—Rh1	126.0 (3)	C17—C19—H18A	109.5
C4—O2—Rh1	125.3 (3)	C17—C19—H18B	109.5
O1—C1—Rh1	174.9 (4)	H18A—C19—H18B	109.5
C36—C31—C32	108.7 (3)	C17—C19—H18C	109.5
C36—C31—P1	115.7 (3)	H18A—C19—H18C	109.5
C32—C31—P1	112.3 (3)	H18B—C19—H18C	109.5
C36—C31—H2	106.5	C17—C18—H19A	109.5
C32—C31—H2	106.5	C17—C18—H19B	109.5
P1—C31—H2	106.5	H19A—C18—H19B	109.5
C35—C36—C31	109.4 (3)	C17—C18—H19C	109.5
C35—C36—H3A	109.8	H19A—C18—H19C	109.5
C31—C36—H3A	109.8	H19B—C18—H19C	109.5
C35—C36—H3B	109.8	C22—C21—C26	112.4 (3)
C31—C36—H3B	109.8	C22—C21—P1	112.2 (3)
H3A—C36—H3B	108.2	C26—C21—P1	112.7 (3)
C36—C35—C34	111.9 (4)	C22—C21—H20	106.3
C36—C35—H4A	109.2	C26—C21—H20	106.3
C34—C35—H4A	109.2	P1—C21—H20	106.3
C36—C35—H4B	109.2	C21—C22—C23	110.9 (3)
C34—C35—H4B	109.2	C21—C22—H21A	109.5
H4A—C35—H4B	107.9	C23—C22—H21A	109.5
C35—C34—C33	111.7 (4)	C21—C22—H21B	109.5
C35—C34—H5A	109.3	C23—C22—H21B	109.5
C33—C34—H5A	109.3	H21A—C22—H21B	108.1
C35—C34—H5B	109.3	C24—C23—C22	111.7 (4)
C33—C34—H5B	109.3	C24—C23—H22A	109.3
H5A—C34—H5B	107.9	C22—C23—H22A	109.3
C32—C33—C34	110.5 (4)	C24—C23—H22B	109.3
C32—C33—H6A	109.5	C22—C23—H22B	109.3
C34—C33—H6A	109.5	H22A—C23—H22B	107.9
C32—C33—H6B	109.5	C23—C24—C25	110.5 (4)
C34—C33—H6B	109.5	C23—C24—H23A	109.6
H6A—C33—H6B	108.1	C25—C24—H23A	109.6
C33—C32—C31	109.5 (3)	C23—C24—H23B	109.6
C33—C32—H7A	109.8	C25—C24—H23B	109.6
C31—C32—H7A	109.8	H23A—C24—H23B	108.1
C33—C32—H7B	109.8	C24—C25—C26	111.3 (4)
C31—C32—H7B	109.8	C24—C25—H24A	109.4
H7A—C32—H7B	108.2	C26—C25—H24A	109.4
C12—C11—C16	116.4 (4)	C24—C25—H24B	109.4
C12—C11—P1	120.7 (3)	C26—C25—H24B	109.4
C16—C11—P1	122.9 (3)	H24A—C25—H24B	108
C13—C12—C11	120.5 (4)	C21—C26—C25	111.6 (4)
C13—C12—C7	112.8 (4)	C21—C26—H25A	109.3
C11—C12—C7	126.7 (4)	C25—C26—H25A	109.3
C12—C7—C8	111.6 (4)	C21—C26—H25B	109.3
C12—C7—C9	111.1 (4)	C25—C26—H25B	109.3
C8—C7—C9	112.1 (4)	H25A—C26—H25B	108
C12—C7—H10	107.3	O3—C2—C3	126.0 (5)
C8—C7—H10	107.3	O3—C2—C5	114.5 (4)
C9—C7—H10	107.3	C3—C2—C5	119.5 (4)
C7—C9—H11A	109.5	C2—C5—H27A	109.5
C7—C9—H11B	109.5	C2—C5—H27B	109.5
H11A—C9—H11B	109.5	H27A—C5—H27B	109.5
C7—C9—H11C	109.5	C2—C5—H27C	109.5
H11A—C9—H11C	109.5	H27A—C5—H27C	109.5
H11B—C9—H11C	109.5	H27B—C5—H27C	109.5
C7—C8—H12A	109.5	C2—C3—C4	126.5 (4)
C7—C8—H12B	109.5	C2—C3—H28	116.8
H12A—C8—H12B	109.5	C4—C3—H28	116.8
C7—C8—H12C	109.5	O2—C4—C3	126.8 (4)
H12A—C8—H12C	109.5	O2—C4—C6	114.5 (4)
H12B—C8—H12C	109.5	C3—C4—C6	118.7 (4)
C14—C13—C12	121.1 (5)	C4—C6—H30A	109.5
C14—C13—H13	119.4	C4—C6—H30B	109.5
C12—C13—H13	119.4	H30A—C6—H30B	109.5
C15—C14—C13	119.9 (4)	C4—C6—H30C	109.5
C15—C14—H14	120	H30A—C6—H30C	109.5
C13—C14—H14	120	H30B—C6—H30C	109.5
